# The CDC’s management and COVID-19 vaccine recommendations during the COVID-19 pandemic: A healthcare worker’s perspective

**DOI:** 10.1371/journal.pone.0354686

**Published:** 2026-08-20

**Authors:** Gail C. D’Souza-Rushton, Uttara Seshu, Liba Blumberger, Laraib Mazhar, Jessica M. Yingst, Jonathan Foulds, William A. Calo, Casey N. Pinto

**Affiliations:** 1 Department of Public Health Sciences, Penn State Center for Research on Tobacco and Health, Pennsylvania State University College of Medicine, Hershey Medical Center, Hershey, Pennsylvania, United States of America; 2 Department of Psychiatry and Behavioral Health, Pennsylvania State University College of Medicine, Hershey, Pennsylvania, United States of America; University of Georgia College of Public Health, UNITED STATES OF AMERICA

## Abstract

The CDC’s COVID-19 pandemic guidelines affected everyone, especially healthcare workers (HCWs) due to the high exposure risks, long hours and stress during the pandemic. Thus, understanding HCWs perceptions and acceptance of these guidelines were crucial to patient outcomes. Our goal was to investigate HCWs views on the Centers for Disease Control and Prevention (CDC) management of the COVID-19 pandemic and vaccine recommendations. Data were collected from an online survey of HCWs in Pennsylvania (August 2022 – February 2023). Eligible participants (n = 580) aged 21 + , reported COVID-19 vaccination status and time of first vaccination. Participants (n = 580) were 83% female and 92% White. From our sample, 71% provided direct care, 57% lived in a suburban area, and 85% had received at least one dose of the vaccine. HCWs had mixed perceptions of the CDC’s management of the COVID-19 pandemic and vaccine recommendations. Positive perceptions of pandemic management highlighted clear communication, a science-driven approach, and an effective crisis response. Criticisms included overregulation of face mask requirements, politicization, and continuously changing policies. Regarding vaccine recommendations, positive perceptions included timely distribution, accessibility, affordability, and clear communication, while criticisms included vaccine mandates, politicization, perceived lack of scientific basis, and inconsistent messaging. Future recommendations for the CDC regarding outbreaks would be to ensure framing of messaging is free from perceived political bias. The CDC should continue to maintain communication with HCWs and focus on providing evidence-based information during future pandemics.

## Introduction

The coronavirus disease (COVID-19) pandemic resulted in an estimated 1.2 million deaths in the United States (U.S.) between March 2020 to February 2025 [1]. As the leading public health agency in the U.S., the Centers for Disease Control and Prevention (CDC) played a pivotal role in providing guidelines for prevention of disease as well as control of the pandemic [2]. The CDCs role included tracking cases, providing up-to-date scientific information, mitigating virus transmission, conducting surveillance and providing public health policy recommendations to control the spread of the disease [3].

The extensive measures and guidelines issued by the CDC impacted everyone, especially healthcare workers (HCWs) as they worked on the frontlines during the pandemic. Face mask requirements, high COVID-19 exposure, long work hours, inadequate access to personal protective equipment (PPE), as well as increased stress and uncertainty regarding the spread of COVID-19 were some of the significant challenges faced by HCWs [4]. Thus, HCWs perceptions and acceptance of the CDC’s guidelines were crucial to patient outcomes and the trajectory of the pandemic.

A qualitative study that assessed HCWs experiences supporting underserved populations in outpatient settings found that lack of coping strategies and organizational support, and burnout were some of the identified themes in the study [5]. Other quantitative studies highlighted that while some HCWs appreciated the rapid dissemination of guidelines and quick vaccine rollouts, others expressed frustrations regarding the communication gaps and changing recommendations that undermined their ability to perform their duties, especially on the frontlines [5–7]. Additionally, studies have highlighted that some HCWs also experienced disproportionately high levels of burnout during the pandemic that distorted their perception towards the pandemic response [7,8].

While studies have reported on the stress faced by HCWs during the pandemic, there is limited research focusing on the nuances of HCWs’ perceptions of the CDCs management of the pandemic and vaccine recommendations. The purpose of this study is to investigate the HCWs perceptions of the CDCs management of the pandemic and COVID-19 vaccine recommendations. Data from this study will be helpful in understanding why HCWs felt positively towards the CDC or criticized them, and how the CDC and other public health entities can respond to public health emergencies in the future.

## Materials and methods

### Study population

Data for this study were collected from a survey that inquired about HCWs PPE strategies during the COVID-19 pandemic. Recruitment was conducted by Penn State researchers and Pennsylvania (PA) Department of Health collaborators to HCWs in PA using multiple methods including direct emails through universities, hospital systems, organizational list-servs, organization specific social media, targeted health care related public social media, flyers, and direct recruitment. A survey link was provided using Research Electronic Data Capture (REDCap), a secure, web-based application designed to support data capture for research studies [9]. Follow-up reminder emails were sent three to four weeks after initial contact that allowed a follow-up email. Potential participants clicked the survey link where they were provided with the summary of research.

Recruitment began on August 22^nd^, 2022 (approximately 1 year and 7 months after HCWs were eligible to receive the COVID-19 vaccine in PA) and ended on February 1^st^, 2023. All participants who worked in the field of health care, lived in PA, were at least 21 years of age, and could read and write in English were eligible for the study. Participants included in the analysis were those who answered the question about their COVID-19 vaccine status, and their perceptions on the CDC’s management and vaccine recommendations of the COVID-19 pandemic. All experimental protocols and methods were approved by and carried out in accordance with relevant guidelines and regulations from the Pennsylvania State University Institutional Review Board (STUDY00017939). All participants provided written informed consent before participating in the survey.

### Survey measures

HCWs were defined as those who “deliver care and services to the sick and ailing either directly as doctors and nurses or indirectly as aides, helpers, laboratory technicians, or even medical waste handlers.” Participants reported demographics and COVID-19 vaccination status by responding to, “Have you personally received at least one dose of a COVID-19 vaccine?” (yes/no).

Participants were then provided with the definition of the CDC: “CDC (Centers for Disease Control and Prevention) is the nation’s public health agency that protects people by preventing and controlling disease, injury, disability, and responds when these health threats arise.”, and asked, “How has your view of the CDC changed based on the agency’s management of the COVID-19 pandemic? My view is...” Response options ranged from more positive to more negative with an option for neutral. If they selected a response other than neutral, they were prompted, “In one or two sentences, can you tell us why your view of the CDC changed based on the agency’s management of the COVID-19 pandemic?”, and could answer with a text response. Similarly, participants were asked about their view of the CDC based on the agency’s COVID-19 vaccine recommendations with the same response options. Those who selected a response besides neutral were asked to provide a text response.

### Data analysis

Means and frequencies were used to describe the sample and quantitative analyses were conducted in R Studio version 4.3.2. Open-ended responses were imported into MAXQDA 2024 for qualitative analysis. Responses labeled “N/A”, one-word answers, or vague responses that did not answer our research question were excluded. Researchers (GD and US) generated an inductive codebook, met to discuss the independently developed codes and finalized the codebook. They first coded 20% of the dataset to establish inter-rater reliability while meeting regularly to refine the codes until a kappa coefficient greater than .80 was achieved. The remaining responses were then independently coded by both researchers.

## Results

Participants (n = 580) were 83% female, 92% White, 3% Hispanic, with a mean age of 46 (SD = 12.66) years. Almost 75% of our sample provided direct patient care, and nearly 39% earned a bachelor’s degree (Table 1). Two themes emerged from our study and had mixed responses. Some participants praised the CDC’s management of the pandemic and COVID-19 vaccine recommendations, while others were critical of it (S1 Table).

**Table 1:** Demographic characteristics of a Pennsylvania sample of HCWs (N = 580).

Characteristics	Overall % (n/N)
Participant characteristics	
Mean age (SD) (n), years	45.79 (12.66)
Woman	83.42 (478/573)
Race	
White	91.90 (533/580)
Black or African American	1.90 (11/580)
American Indian or Alaska Native	0.34 (2/580)
Asian or Pacific Islander	2.24 (13/580)
Other	3.62 (21/580)
Hispanic or Latino	2.62 (15/573)
Provide direct patient care	70.51 (404/573)
Education level	
High school or less	4.08 (23/564)
Associates degree or some college	22.16 (125/564)
Bachelor’s degree	39.18 (221/564)
Master’s or doctoral degree	34.57 (195/564)
Geographic locations of participants	
Urban	13.88 (78/562)
Suburban	57.47 (323/562)
Rural	28.65 (161/562)
Combined household annual income	
$0-19,999	0.43 (2/470)
$20,000–49,999	11.70 (55/470)
$50,000–99,999	31.28 (147/470)
$100,000 or more	56.60 (266/470)
COVID-19 vaccine doses	
Not vaccinated	15.17 (88/580)
At least one dose	84.83 (492/580)

**Theme 1: HCWs had mixed views on the CDC’s management of the COVID-19 pandemic; positive perceptions included continuous communication and transparency, effective crisis response, and a scientific and research-oriented approach while criticisms highlighted changing policies and poor communication, and politicization of the pandemic and overregulation.**

### Positive perceptions on the CDC’s management of the COVID-19 pandemic

#### Continuous communication and transparency.

Many participants felt the CDC ensured continuous communication during the pandemic by providing, “available resources, data, and stats 24/7.” HCWs appreciated that the CDC “simply presented facts and did not openly feed the social media sensitivities.” Some participants reported, “They (the CDC) did a great job of relaying the ongoing information that was changing consistently”.

#### Effective crisis response.

Despite the changing situation and paucity of time, participants also acknowledged CDCs efforts in managing the pandemic. A respondent said, “I believe they (the CDC) handled the pandemic to the best of their abilities given the information they had at the time”. Others also echoed this sentiment, while taking cognizance of the need for improvement during future pandemics saying, “I think the CDC managed the crisis to the best of their ability while recognizing there’s room for improvement”.

#### Scientific and research-oriented approach.

Some participants in the study also expressed confidence in the CDC’s scientific rigor reporting, “CDC was able to quickly view the science and pivot the response accordingly.” Participants also valued the agency’s access to specialists, “They’re at a place to review ALL the data across the country/world AND have access to a variety of specialists.” Other participants appreciated the CDC’s role in combating misinformation with science-based updates. One respondent said, “I appreciated their scientific information shared during a time when misinformation was rampant”.

### Criticisms of the CDC’s management of the COVID-19 pandemic

#### Changing policies and poor communication.

Some HCWs highlighted the lack of clarity in the evolving information regarding COVID-19. One participant reported, “Too often their information was misleading” The CDC’s inconsistent mask mandates also caused confusion among healthcare professionals. One participant said, “The CDC kept suggesting one extreme to the other. Healthcare workers were initially told not to wear any masks and to use a bandana if necessary.”

#### Politicization of the pandemic and overregulation.

Some participants suggested that while scientists at the CDC were unbiased, “political appointees started choosing the messaging in ways that degraded the agency’s credibility in their eyes.” Several participants also believed that “pharmaceutical companies were affecting their (the CDC’s) decisions”. Besides politicization, for some HCWs, the CDC’s regulations seemed to mostly prioritize operational needs, “rules for healthcare workers were changed based on need for staffing in facilities, not based on science or protection of us as healthcare workers.” Other HCWs also highlighted that CDC mandates such as “isolation and prevention forced upon schools and businesses were unnecessary.”

**Theme 2: HCWs had mixed views on the CDC’s COVID-19 vaccine recommendations: positive perceptions included adopting vaccine recommendations based on science, good communication regarding the vaccine, and timely vaccine distribution, while criticisms included minimal scientific foundation, vaccine politicization, unnecessary vaccine mandates, as well as inconsistent vaccine messaging.**

### Positive perceptions on the CDC’s COVID-19 vaccine recommendations

#### Adopting vaccine recommendations based on science.

Many participants appreciated the CDC’s reliance on scientific evidence because, “they (CDC) are still giving us accurate real time direction based on evidence-based criteria.” One participant also emphasized the CDC’s expertise in vaccine recommendations stating, “this is the CDC’s specialty, i trust the science,”.

#### Good communication regarding the vaccine.

Participants appreciated the CDC’s communication regarding the vaccine with one participant saying, “the CDC did great job of relaying the ongoing information that was changing consistently.” Another participant said, “I felt the CDC was in touch quickly regarding the vaccine as soon as one was developed and made available to the public.” Some participants appreciated the CDC’s efforts during the pandemic with one participant saying, “they’ve (CDC) done a lot to educate the public.”

#### Timely vaccine distribution.

Participants also appreciated that the CDC ensured vaccines were available to the public as soon as possible with one participant saying the CDC was, “appropriate to fast-track vaccines and get them out to the public”. Some participants also highlighted that “determining who was vaccinated was very well thought out.” Participants also appreciated the accessibility and affordability and that the CDC had been, “consistent in encouraging vaccination, making it (the vaccine) available, and in continuing to encourage testing and isolation when needed.”

### Criticisms of the CDC’s COVID-19 vaccine recommendations

#### Minimal scientific foundation.

Conversely, participants reported criticisms regarding the vaccine with one participant stating the COVID-19 vaccine was an “Untested, unsafe vaccine rolled out for a virus with a 99% survival rate.” A lot of the participants also felt there was not enough time for the CDC to develop an effective vaccine with one participant highlighting, “Although we were on a time crunch, the vaccine was very much rushed with small population testing before being pushed into emergency approval by the FDA.”

#### Vaccine politicization.

Participants also felt that the COVID-19 vaccine recommendations were influenced by political parties and that “It (vaccine recommendations) appears to change based on political party holding office, elections, etc.” One participant claimed, “We were not given a choice in our facility with the vaccine. it was either get the vaccine or be fired. That is not how America should work, and everything has been made too political.” Some participants also felt that ‘big Pharma’ played a role in the CDC’s decision-making saying, “Having them (the CDC) stand up with the president with companies who make the vaccines and profit off of you getting and using their vaccines, was poor marketing for most Americans.”

#### Unnecessary vaccine mandates.

Many participants also reported concerns regarding vaccine-related mandates from the CDC with one participant saying, “Forcing, bribing, pressuring, coercion of an experimental vaccine, and not acknowledging the onset of the side effects and complications to individuals is unethical and despicable.” Another participant said, “The fact that it was mandated for all age groups, not taking into effect medical background. In my opinion the government and the CDC /NIH/WHO have no business mandating a vaccine. That should be a decision between the patient and the physician.”

#### Inconsistent vaccine messaging.

Some participants also reported on the poor and inconsistent vaccine information shared by the CDC with one participant saying the CDC provided “conflicting information in different time periods.” Another participant felt that the CDC, “did not acknowledge any of the negative effects of the vaccine.” Finally, one participant felt the CDC had, “a horrible distribution method, didn’t reach enough people quickly enough - inability to convince enough people despite being a government organization.”

## Discussion

This study aimed to understand HCWs perceptions on the CDC’s management and vaccine recommendations during the COVID-19 pandemic. The CDC’s response to the COVID-19 pandemic management was perceived with mixed feelings by HCWs. Some HCWs felt positively towards the CDC because they appreciated the CDCs continuous communication and reliance on evidence-based science for recommendations. The CDC provided timely evidence-based updates as well as treatment information on COVID-19. For some participants in our study, the continuous messaging and transparency increased trust during unforeseen circumstances. This is consistent with previous studies where the CDC’s ability to relay evolving recommendations clearly encouraged a positive attitude towards their role during pandemic [10,11].

In comparison, one of the most common criticisms from our study was misinformation. Many HCWs commented on misinformation, criticizing the CDC’s lack of preparation and ability to provide the right information to the public in a timely manner. This finding is consistent with other studies that highlighted the prevalence of misinformation among HCWs during the pandemic [5,12]. Studies have found that frontline workers expressed frustration with the inconsistency of messaging that undermined public trust in the CDC’s expertise [5,12]. Rumors, conspiracy theories, and stigma relating to vaccinations led to public distrust of international health organizations, government organizations and other health entities [12]. Thus, effective communication between the CDC and HCWs is crucial. The CDC, with the help of dedicated marketing and communications experts, should use advanced technology to develop targeted social media campaigns for health messaging and communication. These efforts might help combat misinformation and ensure good communication between the two entities. Some HCWs also emphasized the importance of reviewing recommendations before disseminating inconsistent information to the public. Additionally, other HCWs suggested that the CDC should hold accountability for misinformation and be transparent with the public about the pandemic.

The ever-evolving COVID-19 guidance, while necessary, also seemed disorganized as reported in our study and others [11,12]. Frequent policy changes (e.g., face mask requirements), led to the perception that the CDC lacked preparedness for a large-scale crisis. The CDC was also criticized for over-regulation and political influences on the agency’s decisions. HCWs felt mandates like masking and business closures infringed on personal freedoms while others believed the CDC’s decisions were influenced by pharmaceutical companies, reducing trust in the agency’s credibility [13]. A U.S. study reported HCWs highlighting the CDC’s policies may have been shaped by external political pressures rather than scientific evidence [14]. Consequently, the pandemic management was viewed by some as overly restrictive, contributing to public skepticism about the CDC’s role in balancing public health, science and politics [15]. While political considerations are inherent in governmental decision-making, politicization of the vaccine negatively impacted the pandemic response. [16] Thus, it is crucial for the CDC to prioritize integrity over political influences by considering the use of a marketing campaign in future health crises to ensure public messaging and decision making are transparent and unbiased.

The rapid COVID-19 vaccine development also sparked mixed feelings about the safety and efficacy of the vaccine [17]. Some HCWs were concerned about the speed of vaccine development, while others appreciated immediate availability of vaccines especially for immunocompromised individuals or those with co-morbidities. HCWs were also concerned about vaccine mandates [18]. Although mandates were created to reduce the spread of COVID-19, protect the immunocompromised and prevent worker shortages, some HCWs criticized the mandate [18,19]. Participants reported that the CDC should consider individual medical history particularly for HCWs unable to be vaccinated due to allergic reactions from ingredients in the vaccine. Thus, moving forward the CDC should consider alternative solutions for individuals who cannot physically receive the vaccine, so they may be able to protect themselves, and their job, regardless of vaccine mandates.

Limitations were present in this study. Our study inquired about HCWs perceptions of the CDC’s management of the pandemic and COVID-19 vaccine recommendations and why they felt that way, but no further questions were asked for them to elaborate on their responses. HCWs with strong feelings about COVID-19 management and vaccine recommendations may have been more motivated to respond to open-ended prompts than others who did not feel the same way, which could have led to biased responses. Some HCWs may also have been frustrated with politicians and employers rather than the CDC itself. Additionally, our study had a larger proportion of participants who were White, female and earning over $100,000 which may not be generalizable to the wider U.S. HCW workforce. These demographic characteristics may be associated with certain biases that may have influenced their opinions on the CDC’s management of the pandemic, although travel nursing increased salaries during COVID-19 and while it is not a typical nursing salary, it is likely many nurses were getting COVID-19 bonuses and travel pay so the population earning over $100,000 is likely representative of physicians, advanced practice providers and nurses during COVID-19 [19]. Additionally, our study consisted of a quantitative question and one open-ended question which provided us with one-line answers from our respondents. The timeline of the study also impacts perceptions, and the state (Pennsylvania) may have had some influence although it is a swing state with a mixed urban/rural population that should help overcome some of those biases. This study also had strengths. Our study had a large sample size for our open-ended responses. We queried a broad range of HCWs and not just physicians or nurses or those that solely work in a hospital setting. Finally, this study can provide insight to the CDC and help them understand reasons for why HCWs had both positive perceptions and criticisms towards the CDC so the agency can create better policies for future pandemics.

## Conclusion

This qualitative study with a large sample of Pennsylvanian HCWs suggests that HCWs had mixed feelings towards the CDC regarding their management of the pandemic and COVID-19 vaccine recommendations. Governmental agencies should consider understanding HCWs perceptions towards the CDC so these agencies may work with HCWs to provide continuous communication and information regarding health crises in the future. Understanding positive perceptions can help CDC continue those practices, while understanding criticisms can help the CDC improve their current pandemic practices to inform their decisions for future pandemics.

## Supporting information

S1 Table Qualitative quotes and themes derived from healthcare worker participant. (DOCX)
