# Supplementary material for: The CDC’s management and COVID-19 vaccine recommendations during the COVID-19 pandemic: A healthcare worker’s perspective
Source: PLoS One. 2026 Aug 20;21(8):e0354686. doi: 10.1371/journal.pone.0354686 (PMC13492812; doi:10.1371/journal.pone.0354686)
Supplement: Supplementary file 1 [file pone.0354686.s001.docx]

**S1 Table. Qualitative quotes and themes derived from our HCW participants.**

| **Theme** | **Categories** | **Codes** | **Quotes** |
| --- | --- | --- | --- |
| Theme 1: HCWs had mixed views on the CDC's management of the COVID-19 pandemic; positive perceptions included continuous communication, a science-driven approach, and effective crisis response, while criticisms highlighted overregulation, politicization, and inconsistent policies and preparedness. | Positive perceptions on the CDC’s management of the COVID-19 pandemic | Continuous communication and transparency maintained | *“The CDC offered continuous communication, with available resources, data, and stats 24/7. They simply presented facts and did not openly feed the social media sensitivities.”*  *“I have not experienced a pandemic like this, so to have an organization that had the resources and ability to spread research data was helpful.”*  *“They did a great job of relaying the ongoing information that was changing consistently, with the public.”* |
|  |  | Effective crisis response | *“I believe they handled the pandemic to the best of their abilities given the information they had at the time.”*  *“I believe the CDC made tough ‘on the spot’ decisions to keep Americans as safe as possible without trying to cause too much harm,”*  *“I think the CDC managed the crisis to the best of their ability while recognizing there's room for improvement.”* |
|  |  | Scientific and research oriented | *“They were able to quickly view the science and pivot the response accordingly.”*  *“They’re at a place to receive and review ALL the data across the country/world AND have access to a variety of specialists.”*  *“I appreciated their scientific information shared during a time when misinformation was rampant.”* |
|  | Criticisms of the CDC’s management of the COVID-19 pandemic | Changing policies and poor communication | *“Too often their information was misleading, and that left people to lose trust and for those of us on the frontlines, trying to defend ourselves and the practices.”*  *“Websites were updated overnight with no notification. They took no responsibility for admitting they had misjudged or mischaracterized the disease at any point.”*  *“The CDC kept suggesting one extreme to the other. Healthcare workers were initially told not to wear any masks and to use a bandana if necessary. They went from that extreme to doubling up masks, to the vaccine is necessary and were not forthcoming about any side effects with the vaccine.”*  *“Very disappointed that they were not prepared for an outbreak considering their supposed ‘expertise’ in this arena and decision to shut down the community/businesses, thus causing widespread panic, depression, and economic decline.”* |
|  |  | Politicization of the pandemic and overregulation | *“Believe the scientists did their best to get good data out there quickly, but political appointees started choosing the messaging, and it made me lose a lot of respect for the organization.”*  *“Pharmaceutical companies were affecting their decisions and were biased.”*  *“It was a money grabbing opportunity.”*  *“Rules for healthcare workers were changed based on need for staffing in facilities, not based on science or protection of us as healthcare workers.”*  *“The isolation and prevention forced upon schools and businesses [was] unnecessary.”*  *“Wearing a mask should be OUR choice, not forced on us like the government has done.”*  *“Caused unnecessary hysteria and placed fear in the minds of the US population.”* |
| Theme 2: HCWs had mixed views on the CDC's COVID-19 vaccine recommendations: positive perceptions included adopting vaccine recommendations based on science, good communication regarding the vaccine, and timely vaccine distribution, while criticisms included minimal scientific foundation, vaccine politicization, unnecessary vaccine mandates, as well as inconsistent vaccine messaging. | Positive perceptions on the CDC’s COVID-19 vaccine recommendations | Adopting vaccine recommendations based on science | *“Epidemiological data guided their (CDC’s) recommendations.”*  *“I feel they (CDC) are still giving us accurate real time direction based on evidence-based criteria.”*  *“This is the CDC's specialty, i trust the science.”*  *“Their (the CDC’s) vaccine recommendations are consistent with established science and research.”* |
|  |  | Good communication regarding the vaccine | *“The CDC did a great job of relaying the ongoing information that was changing consistently, with the public.”*  *“I felt the CDC was in touch quickly regarding the vaccine as soon as one was developed and made available to the public.”*  *“CDC kept us informed as to availability of vaccines and numbers of infections.”*  *“They've (CDC) done a lot to educate the public.”*  *“Learn, teach and execute the use of the COVID vaccine.”* |
|  |  | Timely vaccine regulation and distribution | *“Appropriate to fast-track vaccines and get them out to the public.”*  *“They (CDC) did everything possible to have the vaccine as fast as possible.”*  *“Impressed at how quickly the vaccine was available to us.”*  *“Determining who was vaccinated was very well thought out.”*  *“Making it (the COVID-19 vaccine) available and free.”*  *“Consistent in encouraging vaccination, making it available, and in continuing to encourage testing and isolation when needed.”* |
|  | Negative perceptions on CDC’s COVID-19 vaccine recommendations | Minimal scientific foundation | *“Untested, unsafe vaccine rolled out for a virus with a 99% survival rate. Poor outcomes and vaccine injuries, as well as a person's personal beliefs were and are completely disregarded.”*  *“Having worked in research and development of the life sciences, I have a full understanding of how long things take to develop. Although we were on a time crunch, the vaccine was very much rushed with small population testing before being pushed into emergency approval by the FDA. Every government agency should at the very least perform and endorse well, due diligent science.”*  *“There was too much of a rush to push vaccines without significant proof of efficacy.”* |
|  |  | Vaccine politicization | *“It (vaccine recommendations) appears to change based on political party holding office, elections, etc.”*  *“We were not given a choice in our facility with the vaccine. it was either get the vaccine or be fired. That is not how America should work, and everything has been made too political.”*  *“People who control the health care world are being highly manipulated by the government and money. These two motivators are making the CDC less credible in my eyes.”*  *“They already created mistrust with how they handled the pandemic and then also having them stand up with the president with companies who make the vaccines and profit off of you getting and using their vaccines, was poor marketing for most Americans. A lot of myths came out and they did not try to disprove them. They kind of just gave up knowing people will do what they will do.”* |
|  |  | Unnecessary vaccine mandates | *“Forcing, bribing, pressuring, coercion of an experimental vaccine, and not acknowledging the onset of the side effects and complications to individuals is unethical and despicable. Using scare tactics to compel parents to give this to their children is contemptible.”*  *“When nurses were short resources there were many short cuts that made nurses vulnerable to covid and were not supported by agencies or the community. Vaccines are being mandated and people are losing their jobs when the country is on the verge of a depression.”*  *“The CDC strongly recommends vaccines for age groups that are not high risk.”*  *“The fact that it was mandated for all age groups, not taking into effect medical background. In my opinion the government and the CDC /NIH/WHO have no business mandating a vaccine. That should be a decision between the patient and the physician.”* |
|  |  | Inconsistent vaccine messaging | *“It doesn't make sense, nor is it practical. The basic lay person is so brainwashed by it and an educated person can see it doesn't make sense.”*  *“I think that it has been difficult because their recommendations & guidelines are continuously changing.”*  *“Conflicting information in different time periods.”*  *“They (CDC) did not acknowledge any of the negative effects of the vaccine. While the vaccine is overall beneficial, we should be giving ALL the information, not just the info that they want us to hear.”*  *“We should have done better with access to the vaccine for general population in the beginning of the pandemic.”*  *“Horrible distribution method didn't reach enough people quickly enough - inability to convince enough people despite being a government organization.”* |
